# Changes in life satisfaction among unaccompanied asylum-seeking and refugee minors who participated in teaching recovery techniques (TRT)

**DOI:** 10.1186/s13034-023-00595-x

**Published:** 2023-04-18

**Authors:** Anne Kristine Solhaug, Espen Røysamb, Brit Oppedal

**Affiliations:** 1 Department of Childhood and Families, Norwegian Institute of Public Health, PO Box 222, Skøyen, Oslo, 0213 Norway; 2 Promenta Research Center, Department of Psychology, University of Oslo, Oslo, Norway

**Keywords:** Unaccompanied refugee minors, Unaccompanied asylum-seeking minors, Life satisfaction, Teaching recovery techniques, TRT

## Abstract

**Background:**

Unaccompanied asylum-seeking and refugee minors report low life satisfaction and high levels of mental health problems, nevertheless they often do not seek or receive help for their problems. Teaching Recovery Techniques (TRT) is a low-threshold, five sessions intervention developed to reduce distressing war- and disaster-related trauma reactions among children and youth. In this study, we investigate if TRT can contribute to increased life satisfaction among unaccompanied asylum-seeking and refugee minors.

**Methods:**

Asylum-seeking and resettled unaccompanied minors participated in TRT carried out in 15 locations throughout Norway, *n* = 147, mean age = 16.61 (*SD* = 1.80), 88% boys, and 67% from Afghanistan. Life satisfaction was measured by the Cantril Ladder before the intervention, and two- and eight weeks post-intervention. We also included indices of intervention compliance and contextual variables, such as asylum status. We applied a pre- and post-intervention design with linear mixed model analyses to investigate change in life satisfaction.

**Results:**

Life satisfaction significantly increased from pre- to post- intervention, but not for youth whose asylum application had been rejected or who were still awaiting a decision. Indices of intervention compliance were associated with an increase in life satisfaction.

**Conclusions:**

TRT is a potential useful intervention to enhance life satisfaction among unaccompanied asylum-seeking and refugee minors and can be a measure to support positive development among youth at risk for mental health problems. However, TRT initiatives should consider the participant’s stage of asylum process, because harsh immigration policies may overburden the coping capacity. Without further adaptation, TRT seems most useful for youth granted residence. The manual has been revised to include asylum-related stressors.

**Trial Registration:**

ClinicalTrials.gov (16/54,571, registered 30.01.2019).

## Background

Unaccompanied asylum-seeking and refugee minors (URMs) are children and youth who have fled their home-country without the company of any adult legal caretaker [1]. They seek asylum in a foreign country, after being exposed to accumulated negative and traumatic life events, such as experiences of war, extensive social and economic hardship, unnatural death or disappearance of family members, ethnicity-related conflicts, forced recruitment or sexual violence [2]. In their destination country, they are faced with postmigration stressors related to asylum-seeking and acculturation processes [3, 4]. They report more mental health problems than other refugee and non-refugee youth [5], and a review study reported a prevalence between 17 and 85% for post-traumatic stress disorder (PTSD), and 12.7–76% for depression [6]. Despite the frequent mental health problems URMs are burdened with, they underuse specialized mental health services, either because they themselves are unwilling to be referred to such, or because of a lack of capacity and competence within the services [7–9]. Thus, there is a need for alternative, low-threshold interventions that can alleviate their mental distress, that are more acceptable to the youth, and that can reach many URMs in relative short time.

“Teaching Recovery Techniques” (TRT) was developed by the Children and War Foundation [10] to mitigate symptoms of PTSD among children exposed to war and disaster in low resource contexts [11]. However, it is also of importance to get knowledge about the potential of TRT to impact the subjective wellbeing of URMs, such as their life satisfaction. There is a general lack of evidence-based knowledge about life satisfaction among refugees and URMs, particularly about interventions that can enhance this aspect of their wellbeing [12]. Hence, the overall aim of this study is to explore if TRT, an intervention developed to reduce distressing trauma-reactions, may have an additional positive effect by enhancing the life satisfaction of URMs.

### Life satisfaction

Good health and wellbeing for everyone is an overall aim for the United Nations, and is one of the 17 sustainable development goals (goal nr 3) [13]. There are many dimensions of wellbeing, but less clear definitions [14]. According to Diener and Suh [15], “subjective wellbeing consists of three interrelated components: life satisfaction, pleasant affect, and unpleasant affect. Affect refers to pleasant and unpleasant moods and emotions, whereas life satisfaction refers to a cognitive sense of satisfaction with life. Both affect and satisfaction judgments represent people’s evaluations of their lives and circumstances” (p. 200).

In cross-national studies, younger adolescents (11 years) reported better life satisfaction than older adolescents (15 years). This decline with age seemed to be stronger for girls than for boys [16]. Studies of the adult refugee populations also showed that older age was associated with lower quality of life, a more comprehensive indicator of wellbeing, but these studies differed in relation to gender variation [12, 17, 18]. In a study of Iranian refugees in Sweden, males reported lower quality of life compared to women [17], whereas Syrian males reported higher quality of life than their female counterparts [18]. Compared to the general population, refugees reported lower quality of life [18, 19]. In a study from Nigeria, the refugees reported lower quality of life than the non-refugees, however, the protective effect of quality of life on mental illness was the same [19]. In contrast, a Norwegian study found no significant differences comparing levels of life satisfaction among URMs with that of ethnic majority and minority youth, implying that the URMs were satisfied with life, despite higher levels of depression and daily hassles than the two other youth groups [20]. Consequently, better mental health and psychosocial functioning can be some of the benefits of improving wellbeing among asylum-seekers and refugees [19, 21]. Nevertheless, there is a lack of studies evaluating the effect of interventions on wellbeing in these groups [12].

### Teaching recovery techniques (TRT)

TRT is a manualized, group-based intervention based on principles from trauma-focused cognitive behavioral therapy (TF-CBT). In addition to effectiveness studies from low-income countries of war and disaster [22–24], the intervention has been implemented and evaluated among asylum-seeking and refugee children in England [25] and among URMs in Sweden and Norway [9, 26]. Most studies that have evaluated TRT have focused on its effectiveness in reducing symptoms of PTSD and depression [22, 25].

TRT was designed to be relevant for children (> 8 years old) in low-income countries, who are suffering from clinical levels of symptoms of PTSD after having experienced war- and disaster-related traumatic events. The program can be delivered by individuals without a professional psychiatric or psychological background. The TRT comprises five modules. The participants meet in groups (5–15 participants) for one to two hours once a week over five weeks, with two facilitators who have been trained to deliver the intervention in accordance with the manual. In each of the five meetings, the participants are taught and practice various techniques aimed at alleviating the distress associated with the three main PTSD-symptoms groups: hyperarousal, avoidance, and intrusive memories. They are also encouraged to practice the techniques between sessions. The following elements of TF-CBT are included: psychoeducation, relation skills, affective modulation skills, cognitive coping and processing, trauma narrative, in vivo mastery of trauma reminders and enhancing future safety and development.

Many of the proposed change mechanisms of the TRT to alleviate mental distress, may also be related to better wellbeing, e.g., increased coping and perception of control, better understanding of trauma reactions, and enhanced social support from peers and adults affiliated with each TRT-group. These positive adaptations, such as coping and social support, are predictive of life satisfaction and other aspects of positive mental health [17, 20]. Hence, to expand on the current knowledge about the effectiveness of the TRT in reducing mental health problems, we focus on wellbeing outcomes in terms of life satisfaction. This is in line with the dual-continua model, which recognizes that mental illness and positive mental health are related, but distinct dimensions of mental health [27].

### Intervention compliance

The process of transferring an intervention program into the real world consists of several phases, such as the implementation phase, which must be carefully studied to ensure the validity of an intervention [28]. Better implementation leads to better program outcomes, nevertheless, many studies do not include or document implementation indices [28, 29]. Implementation can be studied through facilitator behavior, such as fidelity, the quality of delivery and adaptation of the program, and through participant behavior, such as responsiveness and enthusiasm. These aspects jointly affect the outcome [30]. Previous research on preventive programs has shown that the number of sessions attended and program engagement were associated with better program outcomes [29, 31, 32].

The impact of the techniques and coping strategies that the participants are taught in TRT, are likely to depend on how many of the five TRT sessions they attend, how much they practice the techniques between sessions, as well as their overall contentment with the TRT. We refer to these indices as *intervention compliance*, reflecting the participants’ responsiveness to the intervention. By including indices of intervention compliance, we gain a deeper knowledge about how TRT was received by the participants, and in what ways their engagement with the intervention is related to outcome.

### Asylum status

Most participants in the present study arrived during the so-called 2015 European refugee crisis, characterized by a considerable increase in refugees and migrants coming to Europe, including URMs [33]. This challenged the capacity of the asylum-processing apparatus in many European countries, including Norway, and changed the countries’ immigration policies in direction of more restrictive laws, regulations and practices. In Norway, some of the measures of the new policies contributed to elaborated stress and worries among the asylum-seeking URMs, because many, especially from Afghanistan, were granted a temporary residence permit, which was a permission to stay until the youth reached the age of majority (18 years).

In an intervention study with URMs in Germany, lack of permanent asylum status negatively affected the outcome of TF-CBT by impairing the participants’ feelings of safety [34]. In another study of URMs in Germany, coming from Afghanistan predicted poorer treatment response to a trauma-focused intervention, compared to URMs from African countries [35]. The authors discussed that this was because it was not possible to provide them a safe place for relieving trauma symptoms because of the threat of deportation to Afghanistan [35].

To expand on the discussion from these studies, we speculate if asylum-stress and other strains can take a toll on the motivation to participate in these programs. On the other hand, TRT-participants in the Swedish study, emphasized normalization of trauma reactions and making sense of their past as important aspects of TRT. They also highlighted that they gained a sense of manageability and strategies to cope with their symptoms, and appreciated the social support within the group [26]. As the effect of TRT in different groups of URMs (asylum-seekers and refugees) remains inconclusive, more information is needed about potential variation in the effect of TRT related to the URMs’ context. Asylum-seekers include those who initially launched their claim, those who have their claim rejected and have appealed, and those who have been granted temporary residence permit until they reach the age of majority (18 years). If the asylum claim is approved, the youth is granted residence and are then referred to as a refugee.

### The Norwegian URM context

The care and support for URMs in Norway are structured by governmental laws and regulations and carried out by local public services. All URMs are entitled the same public health care as Norwegian children and youth. However, there are variations in the services provided, especially the mental health services [8]. This was partly explained by professional and organizational variations and disagreement related to the responsibility for URMs’ mental health, and in shortages in capacity to deal with the mental health problems of URMs.

While they are awaiting their asylum interview, which is the basis for processing of their asylum claims, URMs live in special asylum centers located all over the country, separated from ordinary asylum centers for asylum-seeking families and adults. They continue staying in the asylum center during the time it takes to handle their asylum application, and a potential appeal of a rejected claim. As a result of the high numbers of URMs that came to Norway during the European refugee crisis, Norwegian authorities established many new asylum centers on short notice in 2015, but many of these were discontinued already in 2017, due to the more restrictive policies [8].

Resettlement of the URMs who have been granted asylum is regulated by the Directorate of Integration and Diversity, based on available residence places in local municipalities nationwide, where Child Welfare or Refugee services oversee their support. The majority of resettled URMs live in group homes with varying degrees of adult supervision. Only a small proportion of the youngest children are placed in foster homes [36].

### The present study

The present study is part of the Coping among Asylum-Seeking and Refugee Minors-project (CASaRM). CASaRM was commissioned and funded by three governmental directorates responsible for URM asylum centers and resettlement: The Norwegian Directorate for Child, Youth and Family Affairs, the Norwegian Directorate of Immigration, and the Directorate of Integration and Diversity. The Children and War Foundation trained social workers of the asylum centers and resettlement municipalities together with the local health nurses to deliver TRT in accordance with the manual. We established a group of refugee advisors, consisting of three URMs. The group contributed to developing and piloting measures and advised us on recruiting participants to the study.

The overall aim of the present study is to examine if participation in TRT can increase life satisfaction among URMs with war- and trauma-related PTSD symptoms. The more specific objectives are to investigate if

- TRT increases life satisfaction among URMs,
- indices of intervention compliance, such as course evaluation, how often they practice the techniques, and the number of times attending TRT are associated with changes in life satisfaction,
- life satisfaction trajectories following the TRT differ between youth who were granted and not granted residence.

## Methods

Data for the present study was provided by the CASaRM-study, which was conducted between June 2017 and May 2018, in all regions of Norway. The study was approved by the Regional Committee for Medical and Health Research Ethics (2016/355) and registered in ClinicalTrials.gov (16/54,571). Participation was contingent on signed informed consent. Participants who were younger than 16 years gave a verbal consent, and their legal guardian signed the consent form. A security protocol was developed for the project. As we recruited resettlement municipalities and asylum centers, we contacted local child and adolescent mental health services (CAMH), and informed them about the timing of the project, alerting them to the possibility that the researchers or facilitators might identify participating youth in urgent need of treatment. Based on the input from CAMH, a strategy for emergency referrals to their services was agreed upon.

### Recruitment

The target group of the study was URMs speaking Dari, Pashto, Arabic, Tigrinya, or Somali who arrived in Norway in 2015 or 2016. We invited 21 local institutions all over Norway in charge of the care and support of URMs to the CASaRM-project. These included asylum centers and local child welfare or refugee services responsible of coordinating the support for URMs after they received residence. Out of them, 15 institutions agreed to participate in the study. The reason for non-participation was mainly that some asylum centers were discontinued during the time of recruitment due to a reduction in arrivals of asylum-seeking youth following more restrictive immigration policies in 2016 and 2017. The director of each institution made decisions on which of the staff social workers were to be trained as a TRT-facilitator. This person was also the project’s contact person for that institution. One health nurse from the local health services was recruited to carry out TRT in collaboration with the social worker.

In collaboration with the designated contact person, youth in the target group at the recruited institutions of the CASaRM were invited to an information meeting. They were seated in groups according to their mother tongue, around a bilingual research assistant or interpreter. Information about TRT and the CASaRM project was given in Norwegian by a member of the research team and translated to each language group (Dari, Pashto, Arabic, Tigrinya, and Somali). At the information meeting, youth who were interested in the TRT filled out a screening instrument for PTSD-symptoms, CRIES-8 [37]. The members of the research team assisted the youth if necessary. A bilingual research assistant read the questions and explained difficult words in their mother tongue. Youth who scored above the clinical cut-off were invited to the TRT. If URMs with a CRIES-score right below the clinical cut-off requested to take part in the study, because they perceived TRT could be helpful to the problems they were struggling with, we accepted their inclusion.

### Sample

The target group for the present study was 151 URMs who had agreed to participate in the CASaRM-study and had participated in one or more TRT-sessions. At the first data collection, Wave 1 (W1), 143 participants were present, and 127 at Wave 2 (W2) and Wave 3 (W3). There were no differences in age, gender, country of origin, PTSD symptoms, and life satisfaction between the participants who showed up at all data collections, and those who were present at one or two data collections. However, there were significantly more no-shows among participants with granted residence at the W3 data collection, possibly because the youth did not live at the same place within the municipality. Four cases were removed, because of missing data on the life satisfaction measure at all three measurement points. The final sample comprised of 147 URMs. Of these, 85 had responded to the outcome variable at all three measurement points, 41 had responded at two measurement points, and 21 had responded at one measurement point.

Most of the participants were boys (88.4%, *n* = 130), and from Afghanistan (66.7%, *n* = 92), Eritrea (17.4%, *n* = 24), and Syria (6.5%, *n* = 9). Their mean age was 16.61 (*SD =* 1.80). Some had stayed in Norway less than a year (15.4%, *n =* 19), but most of them had been in Norway for one to two years (47.2%, *n* = 58) or more than two years (37.4%, *n =* 46). At W3, more than half of them were refugees (72.1%, *n* = 98) and had been granted asylum and a residence permit. The remaining 27.9% (*n* = 38) were asylum-seekers, either waiting for the initial asylum interview, a response to their application or appeal, or had their asylum application rejected.

### Procedure

Data were collected one week before the first TRT-session (W1), two weeks after the TRT (W2), and eight weeks after the TRT (W3). About a week before the first TRT-session, the research team (research staff and trained bilingual research assistants), visited each site to conduct the data collection. The participants were gathered in groups in a familiar place of their local communities to fill in self-report questionnaires about psychosocial adaptation and mental health issues. The questionnaires were available in Norwegian and the five targeted languages. The bilingual research assistants read the questions in the participants’ mother tongue and explained words and concepts when necessary. This procedure was repeated at the second (W2) and the final data collection (W3). For the current study, it was not possible to apply a randomized controlled trial design, thus no control group was included. We therefore used a pre- and post-design to examine changes in life satisfaction following TRT.

### Measures

Measures and questions that were not already available in the targeted languages (e.g., CRIES-8) were translated by external, professional translators. Subsequently, the bilingual research assistants revised the translations in cooperation with the research team to ensure that the right meaning of the question was conveyed. The main measures (e.g., life satisfaction) were also piloted and discussed with the refugee advisors to get information about their understanding of words and concepts.

Life satisfaction was measured by the *Cantril Ladder* [39]. The measure has been employed in various large-scale surveys with both immigrant and non-immigrant participants, such as The Health Behavior in School-Aged Children (HBSC) – study [40]. The scale is visualized as a ladder, where the top of the ladder represents the best possible life for the participants, and the bottom represents the worst possible life. The participants evaluated their overall life right here and now. Levin and Currie [41] concluded that the Cantril Ladder was a valid and reliable measurement among Scottish adolescents. We used a 1–10 scale version of the Cantril Ladder but transformed the scores into a 0–10 scale for comparison with other surveys applying the Cantril Ladder, such as the HBSC-study ((raw data scale − 1) / 9*10).

*Intervention compliance* was based on three indices:

1. TRT evaluation (Evaluation). At W2, the participants checked how content they were with TRT on a Likert type scale from 1 (not content) to 4 (very content).
2. Participation in the TRT-course (Participation). The TRT facilitators registered the numbers of sessions attended (1–5).
3. Practicing the techniques (Practice). At W2 the participants checked on a Likert type scale how often they practiced the techniques they had learnt during TRT from 1 (never) to 5 (every day).

*Change in life satisfaction*. We used dummies (0/1) for each time-point (W1, W2, W3) to examine change in life satisfaction. By combining and mutually controlling for different time-points, we obtained estimates of the degree of change and significance level, for changes from W1 to W2, from W1 to W3 and from W2 to W3.

*Asylum status* was self-reported. We employed responses at W3, coded as 0 (not granted residence, i.e., rejection or waiting for a decision on their asylum application or appeal) and 1 (granted residence).

*Age* and *gender* were based on self-report and included as co-variates, based on findings suggesting associations of gender and age with life satisfaction [16]. Age ranged from 11 to 23 years. Gender was coded as 1 (boys) and 2 (girls).

*Children’s Revised Impact of Event Scale (CRIES-8*) [37] was applied as a PTSD screening instrument. The measure consists of eight items reflecting intrusive memories and avoidant behavior. The participants reported whether they had never (0), rarely (1), sometimes (3) or often (5) experienced these reactions. A score ≥ 17 has been recommended as inclusion criteria for the TRT [38]. CRIES-8 was validated as a screening instrument for URMs [42], and can be downloaded from Children and War Foundation [10] in various languages, including Norwegian, Dari, Pashto, Arabic, and Somali.

### Statistical analyses

We performed the analyses in R, version 4.0.4 [43] and IBM SPSS version 27. We conducted the linear mixed models using the lme4 package [44], applying Maximum Likelihood (ML) estimation. Drop-out analyses were conducted with student’s *t*-tests. Given the longitudinal nature of the data, we assumed dependency in participants’ scores over time and within the TRT-course they had participated in (random intercept). With no fixed effects added, the intraclass correlation (ICC) was high (ICC = 0.68), suggesting that we should take the clustering effect into account. In all analyses, we adjusted for gender and age.

In the first analysis we investigated a potential increase in life satisfaction from pre- to post-intervention (study aim a). We ran two linear mixed models with the values of all three life satisfaction measurements as outcome. The time-dummies were included as predictors in the first analysis to investigate changes from W1 to W2, and from W2 to W3. Then, we included time-dummies as predictors to examine changes from W1 to W2, and from W1 to W3.

In the second analysis, we examined the association of the three intervention compliance indices (evaluation, practice, participation) with changes in life satisfaction from pre- to post-intervention (study aim b). To conduct these analyses with the linear mixed model approach, we created an interaction term between each compliance index and the dummies. We conducted a linear mixed model for each intervention compliance index and included dummies representing W1 to W2 and W1 to W3.

In the final analysis, we first examined mean level differences at each time point between URMs not granted residence permit and URMs granted residence, by conducting an independent student’s *t*-test. Thereafter, we investigated if the asylum status groups followed different life satisfaction trajectories from pre- to post-intervention (study aim c). For this purpose, we re-ran the models used for study aim a, first for youth not granted residence, and next for youth who had been granted residence.

## Results

### Descriptive statistics

Table 1 shows the correlation matrix and descriptive statistics for total sample. Life satisfaction was highly correlated over time, indicating stability in the construct. The correlation matrix also shows that the intervention compliance indices were low to moderately correlated with each other and were related to life satisfaction in different ways. A positive evaluation of the TRT was associated with better life satisfaction outcome. Participation and practice, however, were not significantly associated with life satisfaction, although these associations became stronger over time. Asylum status was moderately to strongly correlated with life satisfaction. Granted residence was associated with a more positive evaluation of TRT, whereas youth not granted residence participated more often. Practice was not related to asylum status. The co-variates age and gender were not significantly associated with the main variables, except that older age was associated with a more positive evaluation of the TRT, and females reported better life satisfaction at W2.

**Table 1:** Correlation matrix and descriptive statistics for total sample

	*n*	*M*	*SD*	1	2	3	4	5	6	7	8	9
1. Life Sat W1	108	4.34	2.79	-								
2. Life Sat W2	125	4.77	2.52	0.56^**^	-							
3. Life Sat W3	126	5.12	2.76	0.68^**^	0.80^**^	-						
4. Evaluation W2	121	2.90	0.78	0.06	0.23^*^	0.31^**^	-					
5. Participation	147	3.89	1.30	− 0.15	− 0.09	0.02	0.20^*^	-				
6. Practice W2	124	2.88	1.02	0.02	0.18	0.16	0.36^**^	0.18^*^	-			
7. Asylum W3	136	-	-	0.42^**^	0.61^**^	0.60^**^	0.21^*^	− 0.19^*^	0.01	-		
8. Gender	147	-	-	0.05	0.19^*^	0.11	0.04	− 0.09	0.04	0.23^**^	-	
9. Age	142	16.61	1.80	− 0.17	0.14	0.04	0.28^**^	0.05	0.10	0.28^**^	0.27^**^	-

*Note*: Life Sat = life satisfaction (transformed); Asylum = asylum status (dummy coded)

W1 = Wave 1; W2 = Wave 2, W3 = Wave 3

*p <0.05, **p <0.01

**Fig. 1:**
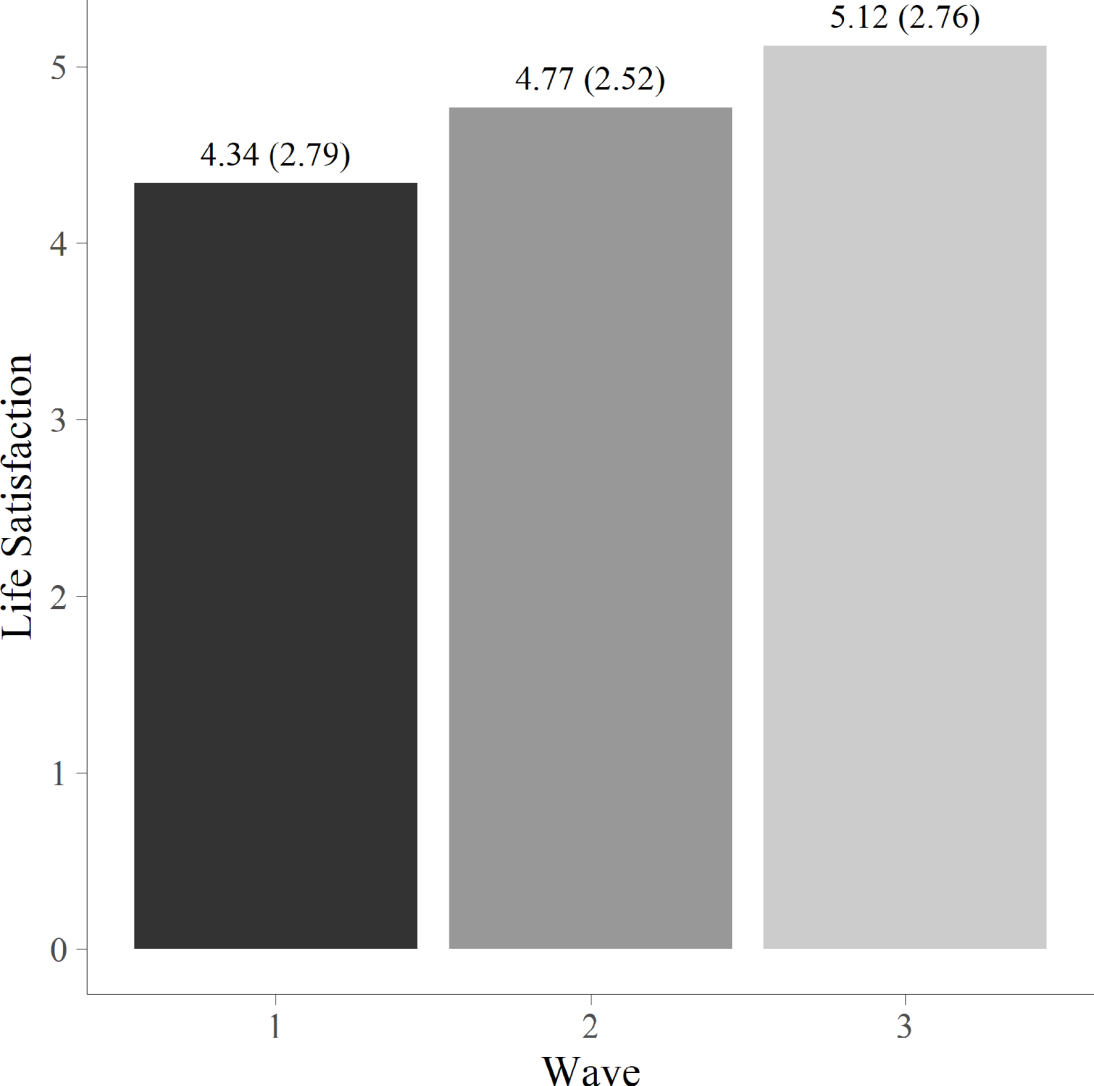
Mean level of life satisfaction at each wave for the total sample

### Change in life satisfaction over time

Figure 1 shows that there was an increase in the mean level of life satisfaction from W1 to W2, and a continuous increase to W3. The results from the linear mixed models show that the change from W1 to W2 was significant *b* = 0.67, 95% CI [0.27, 1.08], *p* < .01, Cohen’s *d* = 0.25. Even if the subsequent increase from W2 to W3 was not significant, *b* = 0.31, 95% CI [-0.07, 0.70], *p* = .11, the changes in level of life satisfaction from W1 to W3 appeared substantially stronger than from W1 to W2, *b* = 0.99, 95% CI [0.58, 1.40], *p* < .01, with a Cohen’s *d* of 0.37. In summary, the results showed a significant increase in life satisfaction from W1 to W2, the first post-intervention test, with a retained improvement from W1 to the final post-intervention test at W3.

### Compliance indices and life satisfaction

**Fig. 2:**
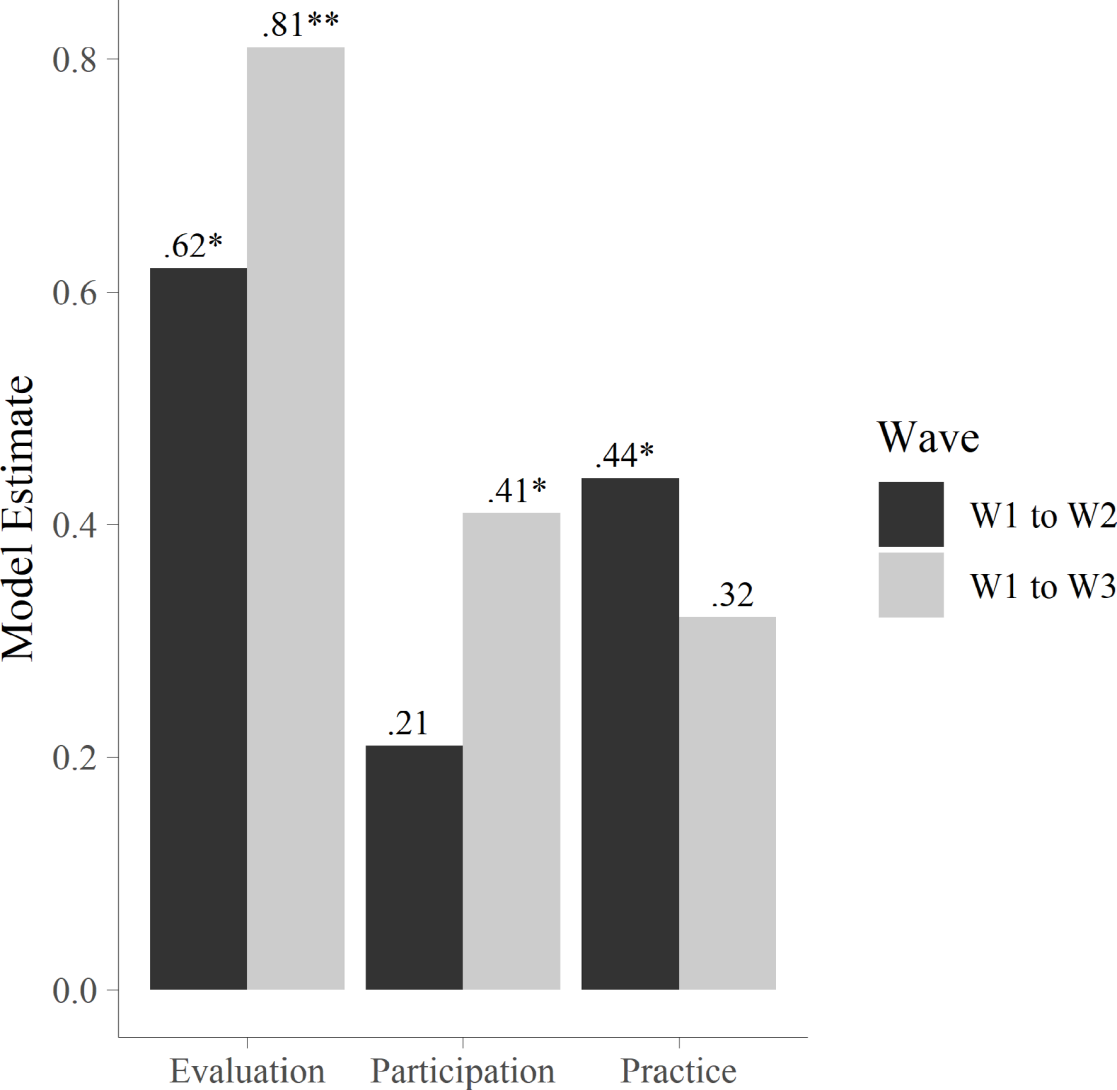
Effects of intervention compliance on life satisfaction change. *Note*: Unstandardized estimates. The figure shows how the indices of intervention compliance interact with changes in life satisfaction from W1 to W2, and from W1 to W3. *p <0.05, p** <0.01

The results showed that there was a significant effect of *evaluation* on change in life satisfaction from W1 to W2; *b* = 0.62, 95% CI [0.10, 1.13], *p* = .02, Cohen’s *d* = 0.23, and from W1 to W3; *b* = 0.81, 95% CI [0.27, 1.34], *p* < .01, the Cohen’s *d* = 0.30. There was also a significant effect of *participation* on change in life satisfaction from W1 to W3, *b* = 0.41, 95% CI [0.08, 0.73], *p* =  .01, Cohen’s *d* = 0.15. Finally, *practice* was significantly associated with increases in life satisfaction from W1 to W2, *b* = 0.44, 95% CI [0.04, 0.84], *p* = .03, the Cohen’s *d* = 0.16. Figure 2 shows variation in the significant associations between the three intervention compliance indices and changes in life satisfaction from pre- to post-intervention. The strongest associations were between evaluation and changes in life satisfaction from W1 to W3. While participation was only significantly associated with changes from W1 to W3, practice was significantly associated with changes from W1 to W2.

### Life satisfaction by asylum status

In Table 2, we present mean, standard deviation and the results of independent sample *t*-tests for youth granted residence and youth not granted residence. The Table shows that there were significant mean level differences between the two groups at each time point. The differences between the groups at all time points showed large effect sizes, Cohen’s *d* ranging from − 1.05 (W1) to -1.66 (W2).

The results of the linear mixed models showed that youth with granted residence had a steady and significant increase in life satisfaction, from W1 to W2, *b =* 0.91, 95% CI [0.41, 1.41], *p <* .01, Cohen’s *d* = 0.34. However, the increase from W2 to W3 was not significant, *b =* 0.28, 95% CI [-0.23, 0.78], *p =* .28. The increase in life satisfaction from W1 to W3 was also significant, over and above the change from W1 to W2; *b =* 1.19, 95% CI [0.68, 1.69], *p <* .01. Cohen’s *d* = 0.44. The average change in life satisfaction from pre- to post-intervention for youth who had not been granted residence was not significant.

**Table 2:** Descriptive statistics and independent sample t-tests for granted residence and not granted residence

	Residence		No Residence		*t*	*p*	Cohens’ *d*
	M	SD	M	SD			
Life Sat W1	5.01	2.69	2.31	2.10	−4.49	< 0.001	−1.05
Life Sat W2	5.85	2.03	2.51	1.93	−8.28	< 0.001	−1.66
Life Sat W3	6.19	2.25	2.60	2.13	−8.33	< 0.001	−1.62

*Note*: Mean parameters for each group are shown for residence (*n* = 100) and no residence (*n* = 38). Also, we present the results of three independent Student’s t−test, comparing the mean scores across the two groups. Degrees of freedom for W1, W2, and W3 were 97, 117, and 123.

## Discussion

The overall aim of this study was to investigate if participation in TRT, a low-threshold, group-based trauma-focused intervention, could contribute to increased life satisfaction among URMs in Norway. Hence, the study is important as it contributes to the much-needed knowledge about the effectiveness of psychological interventions on wellbeing outcomes [12]. Another strength of the study is that it is based on information from a relatively large and presumably representative nationwide sample of URMs with high levels of symptoms of PTSD in various stages of the asylum process.

### Increase in life satisfaction from pre- to post-tests

There was a significant positive change in life satisfaction for the total sample. This finding is promising, considering the sample of URMs with clinical range symptoms of PTSD, a history of traumatic exposure and disrupted social ties, and a stressful everyday situation. In the present study, we also found that the increase in life satisfaction from pre-intervention to the first post-intervention test persisted until the subsequent follow-up test. This is consistent with some TRT-studies examining mental health outcomes [23, 45], but contrary to a study of asylum-seeking children in UK, where the children showed a reduction in symptoms of PTSD, but this was not maintained at follow-up [25]. These findings have clinical significance because even though the youth have high levels of PTSD-symptoms, life satisfaction can improve. This shows that life satisfaction and other aspects of positive mental health are important to target in clinical practice and through interventions with distressed children and youth, as indicated by the dual-continua-model [27].

The positive change in life satisfaction following participation in a low-threshold intervention is in line with one of the few studies investigating life satisfaction among URMs. Participants and non-participants in an expressive art therapy intervention showed different trajectories of life satisfaction, symptoms of PTSD, and mental distress [46]. This was most evident for the life satisfaction trajectory, showing that psychological interventions can lead to different trajectories of mental distress, symptoms of PTSD, and life satisfaction, and signals why it is important to study both illbeing and wellbeing, both for clinical and theoretical purposes [46].

Previous TRT-studies have suggested possible change mechanisms, such as increased sense of coping and better control of the trauma reactions, social support within the TRT-group and normalization of their reactions [22, 26]. We suggest that these mechanisms can explain the observed changes in life satisfaction. Future studies, however, should inspect change mechanism in the intervention, and identify the techniques that the children consider to be the most helpful.

### Intervention compliance

Overall, the results showed that a positive evaluation of TRT, number of times participating and practicing, were associated with enhanced life satisfaction over time, but in different ways. These findings substantiate that the changes in life satisfaction can be related to participation in the TRT, and not merely other circumstances, like the passage of time or better adaptation.

The results showed that youth who evaluated the TRT more positively, had a stronger increase in the level of life satisfaction compared to youth who were less content, two thirds of a step at Cantril ladder. This association can be bi-directional; youth with better life satisfaction might be more likely to be content with the activities they engage in, as noted by Lykken and Tellegen [47]. However, a happy-go-lucky personality cannot fully explain the changes in life satisfaction associated with a positive evaluation of TRT.

We also found a significant association between the number of times participating and changes in life satisfaction. Altogether, the slope increased with almost a half-step on the Cantril ladder from the first and to the final measurement point, implying that participating more often contributed to increased life satisfaction. Similar dose-response effects between participation and a positive outcome were not found in a review of wellbeing interventions [48] and in a TRT-study among URMs in Sweden [26]. Why our results differ from these studies is beyond the scope of this study, but it is likely that participation leads to better understanding of the techniques, reflects a sense of coping and stronger social networks.

Practicing the techniques was associated with an increase in life satisfaction from W1 to W2, almost a half-step on the Cantril Ladder. Contrary to what was the case with evaluation and participation, this effect did not persist over time. It is, however, likely that most of them practiced the techniques while participating in the TRT, but not so much after the TRT had finished. As practicing seems to be important for youths who continue struggling with mental trauma-reactions, we suggest that the TRT instructors organize booster sessions, where they repeat the techniques and encourage the participants to persist using them. Future studies could investigate if TRT booster sessions have further effect on mental health outcomes.

### Asylum status

Based on advice from our refugee advisors, we included asylum status to gain a deeper understanding of life satisfaction and TRT-outcomes among URMs. There was a significant and large difference in life satisfaction between youth granted residence and not granted residence at each measurement point. This finding is of clinical and practical relevance because it points to the importance of distinguishing between the stressors for asylum-seekers and refugees when planning interventions and treatment programs, as well as for future research studies. The TRT-manual has been revised to better address these differences.

Youth who had not been granted residence, as they were either waiting for a decision on their case, had appealed a rejection, or were waiting for deportation when turning 18 years, did not report significant change in life satisfaction after TRT. Although not significant, it is noteworthy that their mean scores increased over time, and not decreased, considering their situation. As many of them lack hope and feel insecure about their future, this may point to a protective effect of the intervention. However, on-going threat can influence treatment outcomes, and is necessary to acknowledge while working with cognitive restructuring in trauma-focused treatments [49]. A therapeutic goal could be to help the youth enhance a sense of control and efficacy, even though the possibility for influencing the situation is limited [49].

As a group, youth who had been granted residence showed increased life satisfaction from pre- to post intervention, and this effect persevered between the two follow-up-tests. At the final measurement point, the mean level of life satisfaction in this group was close to the scores for the Norwegian population (7.37) [50]. Youth who have been granted residence probably have more surplus energy and motivation to participate in activities such as the TRT, and are better to concentrate on new learning tasks, to engage in social relations, and remember topractice the techniques between sessions. We cannot, however, not rule out that better adaptation and other unmeasured events also contributed to increase their life satisfaction.

Overall, these findings are in accordance with studies comparing wellbeing outcomes among individuals with different asylum status, or whose asylum status changed from temporary to permanent stay [51, 52], and studies emphasizing the role of the asylum process on intervention effects [34, 35]. This implies that the uncertainty or the fear associated with the asylum status is a here-and now stressor, which must be targeted when developing psychosocial interventions for this group. The Children and War Foundation- UK has recently made a version of the TRT-manual with techniques to target stressors associated with the uncertainties of the asylum-seeking situation in response to feedback from CASaRM-researchers and other TRT-research teams. The revised TRT-manual includes a session addressing living with uncertainty, both the uncertainty about life back home and the uncertainty about their future in the new country, by focusing on worries and relaxation-exercises [53]. To our knowledge, so far this manual has not been evaluated and this should be a target for future TRT-studies.

### TRT in a stepped care model

The results from the present study show that we have identified a beneficial intervention in terms of increased life satisfaction, adding to other studies suggesting that TRT could be a useful program in a stepped care model for URMs and other asylum-seeking and refugee children [9,26]. A stepped care approach for URMs could be based on trauma sensitive care [54] at the asylum center and in the resettlement municipalities. The social workers can then identify individuals in need of more help. In this model, TRT can serve as the next step. For URMs with severe mental health problems or who do not benefit from the TRT, additional treatment in the CAMH must be provided. The life situation can be complex, and it is thus necessary with flexible approaches in the work with URMs. No single program can target the reciprocal and interrelated psycho-social and mental health needs of refugees, and the actors in the field must coordinate their work and establish an integrated response to the complex need of the refugee population [55]. Nevertheless, TRT can serve as a low-threshold intervention that can ease the pressure in CAMH. It can be integrated into the routine public health services or implemented at times with high arrivals of asylum-seekers and refugees, as in the current project.

### Limitations

The major limitation in this study is the lack of the control condition, due to real-life barriers affecting the data collection. The funding, time frame, and the discontinuance of some of the asylum centers in the project resulted in an insufficient control group. This implies that we cannot make final conclusions about the likelihood that the increase in life satisfaction among URMs following TRT is caused by the intervention or is due to other unobserved circumstances.

Furthermore, we have included intervention compliance indices to investigate if aspects of the intervention are related to change in life satisfaction. We focused on participant responsiveness, but we have not fully accounted for facilitator behavior, which is also an important part of program implementation [30]. This could have further increased our conclusions and provided us with important knowledge about the implementation of the intervention. Potential variation in intervention compliance between the two asylum conditions would also been useful to explore further, but there were too few participants without a residence permit to conduct meaningful interaction analyses.

The sample consists of multi-ethnic youth with different languages and cultural backgrounds, living in various residence settings in different parts of Norway, and with clinically high levels of PTSD-symptoms. Most participants, however, were males from Afghanistan, reflecting the URM-population in Norway at that time [56]. However, even if the sample is presumably representative for the URM population at the time, the overrepresentation of Afghan youth may limit the generalization of the findings beyond this national group. This group was more likely to be granted temporary residence permit, and thus less likely to be granted residence permit than the other national groups [57].

We also included self-report questionnaires in a sample with varied language competence, especially with respect to reading in their mother tongue, which might affect how they filled in the questionnaires and how well they understood the questions. Some participants had years of schooling before coming to Norway, others had not. However, everyone attended Norwegian schools or participated at the municipal education program for refugees, and had started learning to speak, read and write in Norwegian. To accommodate variation in reading experience, the questionnaires were available in Norwegian and in the participant’s mother tongue. Moreover, the trained, bilingual research assistants read the questions in the mother tongue and offered explanations of difficult words or concepts when necessary.

Future studies should aim to implement state of the art research design, and include more comprehensive indices of intervention compliance, including facilitator behavior, and if possible, observational data when evaluating the implementation and intervention effectiveness.

## Conclusion

In this study, we have identified a beneficial intervention to increase life satisfaction among URMs. The participants reported better life satisfaction over time, which was further increased if they evaluated the intervention positively, participated more, and practiced more. The delivery of TRT without any adaptation to the asylum-context, seems to be most suitable for youth granted residence. Further evaluations with higher level of evidence are required, but this study serves as an initial investigation of initiatives aiming to increase life satisfaction. These kinds of interventions are lacking in the URM knowledge base - yet are highly needed. URMs report severe distress and low life satisfaction yet many are not gaining or seeking help. A low-threshold intervention like the TRT can be a part of a better coordinated service aiming to improve overall wellbeing. However, the results from this study also point to a further strengthened effort to ease the distress and low life satisfaction among youth with rejection or waiting for the outcome of the asylum claim. This includes measures ranging from even better tailored interventions delivered in the appropriate stage of migration, and structural change in the asylum policy for URMs.

## Data Availability

The datasets generated and/or analyzed during the current study are not publicly available because the participants have not consented to data sharing.

## Abbreviations

CASaRM: Coping among Asylum-Seeking-and Refugee Minors
CAMH: Child and Adolescent Mental Health Services
CRIES: Children Revised Impact of Event Scale
PTSD: Post-traumatic stress disorder
TF-CBT: Trauma-focused cognitive behavioral therapy
TRT: Teaching Recovery Techniques
URMs: Unaccompanied asylum-seeking and refugee minors.
